# Genetic Transformation and Hairy Root Induction Enhance the Antioxidant Potential of *Lactuca serriola* L.

**DOI:** 10.1155/2017/5604746

**Published:** 2017-08-01

**Authors:** Mohamed A. El-Esawi, Amr Elkelish, Hosam O. Elansary, Hayssam M. Ali, Mohamed Elshikh, Jacques Witczak, Margaret Ahmad

**Affiliations:** ^1^Botany Department, Faculty of Science, Tanta University, Tanta, Egypt; ^2^UMR CNRS 8256 (B2A), IBPS, Université Paris VI, Paris, France; ^3^Botany Department, Faculty of Science, Suez Canal University, Ismailia, Egypt; ^4^Floriculture, Ornamental Horticulture and Garden Design Department, Faculty of Agriculture (El-Shatby), Alexandria University, Alexandria, Egypt; ^5^Botany and Microbiology Department, College of Science, King Saud University, Riyadh 11451, Saudi Arabia; ^6^Timber Trees Research Department, Sabahia Horticulture Research Station, Horticulture Research Institute, Agriculture Research Center, Alexandria, Egypt; ^7^Department of Biology, Xavier University, Cincinnati, OH, USA

## Abstract

*Lactuca serriola* L. is a herbaceous species, used for human nutrition and medicinal purposes. The high antioxidant capacity of *L. serriola* indicates the possibility of enhancing its edible and health potential by increasing the flavonoid and phenolic contents. The present study aimed at enhancing the production of phenolics and flavonoids by hairy root cultures in *Lactuca serriola* transformed with *Agrobacterium rhizogenes* strain AR15834 harbouring the *rolB* gene. The genetic transformation of *rolB* in transformed roots was validated, and *rolB* expression level was evaluated using real-time qPCR analysis. Expression levels of flavonoid biosynthesis genes (CHI, PAL, FLS, and CHS) were assessed in the hairy and nontransformed roots. Results showed higher expression levels in the transgenic roots than in the nontransformed ones (*p* < 0.01). Transgenic hairy roots exhibited a 54.8–96.7% increase in the total phenolic content, 38.1–76.2% increase in the total flavonoid content, and 56.7–96.7% increase in the total reducing power when compared with the nontransgenic roots (*p* < 0.01). DPPH results also revealed that the transgenic hairy roots exhibited a 31.6–50% increase in antioxidant potential, when compared to normal roots. This study addressed the enhancement of secondary metabolite biosynthesis by hairy root induction in *L. serriola*.

## 1. Introduction

Cellular reactive oxygen species (ROS) may arise during the process of mitochondrial oxidative metabolism or due to interactions with some agents like xenobiotics [1, 2]. Oxidative stress occurs because of the imbalance between ROS production and antioxidant defence activity [1, 2]. Oxidative stress causes ROS-mediated macromolecular damage which results in severe diseases [1–4]. Phenolics and flavonoids are natural antioxidants and effective ROS scavengers [2], widely distributed in plants. Therefore, human diet plants have a key role in disease prevention and maintaining health [2].

*Lactuca serriola* L. of the family Asteraceae is a herbaceous species [5, 6], cultivated in Europe, Siberia, Pakistan, Iran, and India [7]. The plant is used for human nutrition and in various medicinal purposes as sedative, expectorant, purgative, cough suppressant, antiseptic, diuretic, and antispasmodic [7]. The plant is rich in lactucarium and comprises a wide range of mineral nutrients, vitamins, natural antioxidants, flavonoids, and phenolics [7]. Pharmacological evaluation of the plant extract showed its anti-inflammatory, anticarcinogenic, and antioxidant potential due to its high phenolic content that revealed effective free radical scavenging activity [7–10]. Though the phytochemical level is relatively less in *Lactuca serriola*, its high antioxidant capacity indicates the possibility of enhancing its edible and health potential upon increasing the flavonoid and phenolic content. The enhancement of secondary metabolites through field cultivation has many defects such as slow growth and low and variable yield due to the environmental and biotic factors [11]. Therefore, hairy root culture has been developed as a more efficient alternative biotechnological technology for secondary metabolite synthesis, regardless of environmental, seasonal, and climatic variations [11, 12]. In vitro hairy roots formed by genetic transformation have been efficiently utilized for the synthesis of higher levels of secondary metabolites due to their biochemical and genetic stability as well as their fast growth in media without phytohormones [11, 13].

Several studies have demonstrated that *Agrobacterium rhizogenes*-mediated transformation with root locus (*rol*) genes induces secondary metabolite biosynthesis in transgenic roots by activating biosynthetic genes [14–18]. Few earlier studies on the hairy root induction of *Lactuca sativa* have been reported [16–18]. However, the secondary metabolite synthesis by *Lactuca serriola* hairy roots has never been reported yet. Our current study aimed to assess and enhance the production of phenolics and flavonoids by hairy root cultures, for the first time, in *Lactuca serriola* transformed with *Agrobacterium rhizogenes* strain AR15834 harbouring the *rolB* gene. The genetic transformation of the *rolB* gene in transgenic roots was validated, and *rolB* expression level was evaluated using real-time quantitative PCR analysis (RT-qPCR). We also evaluated the expression levels of four flavonoid biosynthetic genes (chalcone isomerase (CHI), chalcone synthase (CHS), phenylalanine ammonia-lyase (PAL), and flavonol synthase (FLS)) of transformed and nontransformed roots. Additionally, we estimated the total flavonoid and phenolic contents of hairy and nontransgenic roots of the plant. Finally, we assessed the antioxidant and cytotoxic activities of transformed and normal root extracts of *Lactuca serriola*.

## 2. Material and Methods

### 2.1. Plant Material and Bacterial Strain

*Lactuca serriola* L. seeds were received from the Centre for Genetic Resources (CGN) in the Netherlands. Seeds were first sterilized using 5% NaClO for 5 min, washed 5 times in sterile H_2_O, and then grown on 1/2 MS medium [19, 20] for 16 h light/8 h dark at 24°C.

*Agrobacterium rhizogenes* strain AR15834 harbouring the *rolB* gene was used for transformation and was cultured on liquid Luria-Bertani (LB) media in darkness at 28°C for 48 hours with shaking.

### 2.2. Transformation, Hairy Root Induction, and Root Biomass

Cotyledonary leafy explants of 2-week-old seedlings were cut and infected with the bacterial suspension (OD_600_ = 0.5) for 10 minutes, dried with an autoclaved filter paper, and cultivated on MS media in darkness at 26°C for 4 days. The explants were then transferred onto fresh media of the same constituents and supplemented with cefotaxime. The formed roots were then transferred onto liquid Woody Plant Media (WPM) lacking growth regulators. Cultures were put on a rotary shaker at 80 rpm in darkness. Subcultures were performed every 30 days (0.5 g fresh root biomass was transferred onto new media). Hairy root biomass (fresh and dry weights) was estimated after 30 days of culture. For each hairy root line, 3 flasks from 3 successive subcultures were utilized. The hairy roots showed stability with regard to a root biomass increase. The untransformed (control) roots were also grown on the same media.

### 2.3. Molecular Analysis of Hairy Roots by PCR

To validate transformation, total genomic DNA was prepared from transgenic roots (5 survived lines) and nontransformed roots (negative control) using the DNeasy Plant kit from Qiagen in UK, following the manufacturer's procedures. Plasmid DNA was also purified from *A*. *rhi*z*ogenes* strain AR15834 and used as a control. A primer pair designed by Skała et al. [21] was used for amplification of the *rolB* gene (a fragment size of 386 bp; Table 1). Additionally, to confirm the correct transformation of hairy roots without bacterial contamination, PCR amplification included the *vir*G gene (Table 1) [21]. Amplification was conducted in reactions of a final volume of 25 *μ*l (1.5 *μ*l of each primer (50 ng/*μ*l), 2 *μ*l of DNA (25 ng/*μ*l), 12.5 *μ*l of master mix, and 7.5 *μ*l of dist. H_2_O. PCR amplification programme was set up: 3 min at 95°C; 33 cycles of 30 sec at 95°C, 30 seconds at 55°C, and 2 min at 72°C; and then 3 min at 72°C. PCR products amplified were then visualized on 1.2% agarose gel and photographed.

### 2.4. Expression Analysis of the *rolB* Gene and Flavonoid Biosynthetic Genes

Real-time quantitative PCR (RT-qPCR) was performed to evaluate the expression level of the *rolB* gene in transgenic roots as well as changes in expression levels of four flavonoid biosynthesis genes (CHI, PAL, FLS, and CHS) of transformed and nontransformed roots. Total RNA was prepared from the transformed (5 lines) and nontransformed roots using the RNeasy Plant Mini kit, and cDNA synthesis was done using the Reverse Transcription kit (Qiagen). RT-qPCR was done in triplicates with the QuantiTect SYBR Green PCR kit from Qiagen. PCR amplification programme used was set up: 95°C for 5 min and 35 cycles of 95°C for 30 sec, 57°C for 30 sec, and 72°C for 2 min. The primers of genes analyzed are shown in Table 1. Analysis of the melting curve was then used to test the amplification specificity. Gene expression level was normalized to ubiquitin (UBQ1) housekeeping gene level [22] and calculated using the 2^−∆∆Ct^ method.

### 2.5. Extract Preparation

To conduct antioxidant assays, extracts from the transgenic and nontransformed roots of *Lactuca serriola* were prepared. For each type, 5 grams of dried plant material was soaked in 100 ml methanol in a sonication bath for 2 hours. The extracts were then filtered and concentrated. The concentration of extracts used in antioxidant assays is 50 mg/ml sterile water.

### 2.6. Total Phenolic Content (TPC)

TPC of the transformed and nontransformed extracts was estimated following the Folin-Ciocalteu reagent assay [18, 23] with minor modifications. In brief, 4 *μ*l of plant extract was added and mixed with 98 *μ*l of diluted Folin-Ciocalteu solution and kept at 26°C for 10 min; then, 98 *μ*l of 5% Na_2_CO_3_ was added to the mixture and kept for 2 hours at 26°C. Solution absorbance was then determined at 725 nm. TPC was represented as a gallic acid equivalent. Triplicate analyses were done.

### 2.7. Total Flavonoid Content (TFC)

TFC of the transformed and normal extracts was estimated following the reported assay [24] with minor modifications. In brief, 4 *μ*l of each extract was mixed with a solution (10 *μ*l of 1 M potassium acetate, 10 *μ*l of 10% AlCl_3_, and 176 *μ*l of H_2_O) and kept at 26°C for 30 min. Mixture absorbance was then determined at 405 nm. TFC was represented as a quercetin equivalent.

### 2.8. Total Reducing Power (TRP)

TRP of the transgenic and nontransformed extracts was estimated following the reported method [24] with minor modifications. Twenty microliter of each extract was added to 490 *μ*l 1% potassium ferricyanide and 490 *μ*l 0.2 M phosphate buffer and mixed and kept for 20 min at 50°C. 500 *μ*l of 10% TCA was then added to the solution and centrifuged; then, 500 *μ*l of the upper layer was mixed with 100 *μ*l of 0.1% ferric cyanide in a new Eppendorf tube. The solution absorbance was then determined at 630 nm. TRP was represented as an ascorbic acid equivalent. Analyses were done in triplicate.

### 2.9. 2,2-Diphenyl-1-picryl-hydrazyl (DPPH) Free Radical Scavenging Assay

DPPH of the transformed and untransformed extracts was estimated following the DPPH protocol [18]. Ascorbic acid was utilized as a control. Triplicate analyses were conducted.

### 2.10. Cytotoxicity Screening Assay

HepG2 human liver cancer cells (obtained from VACSERA, Egypt) were utilized for the cytotoxicity test of root extracts of *Lactuca serriola*. Cell viability was recorded with the MTT method [25]. Human cells were added to each well of 96-well plates and were subjected to various concentrations of transformed and nontransformed extracts of the transgenic line of *rolB2* (12.5, 25, 50, 100, and 200 *μ*g/ml) for 48 hrs. Cells were incubated with MTT (20 *μ*l per well) at 37°C for 2 hours. DMSO was then used. Optical density was measured at 492 nm. Cell growth inhibition percentage was estimated as reported by Chung et al. [11]. Analyses were done in triplicate.

### 2.11. Data Analysis

Results were represented as means with standard deviation. ANOVA analysis and test for significant difference (*p* < 0.05) were done using SPSS software.

## 3. Results and Discussion

### 3.1. Transformation, Hairy Root Induction, and Root Biomass

**Figure 1:**
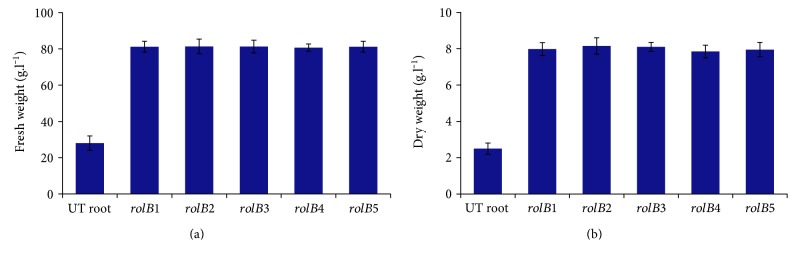
Fresh and dry weights of the untransformed root (UT root) and the 5 transgenic hairy root lines after 30 days in liquid media. (a) Fresh weight (g) of biomass per liter; (b) dry weight (g) of biomass per liter.

*Lactuca serriola* L. was transformed with *A. rhizogenes* strain AR15834 containing the *rolB* gene. Approximately, 250 explants were transformed, and transformation efficiency of producing hairy roots was high (74%). However, only five transformed lines survived till the maturity stage. Similar high transformation efficiency with *A. rhizogenes* AR15834 was found in *Tribulus terrestris* L. by Sharifi et al. [26]. Efficiency of transformation and hairy root induction relies on *A. rhizogenes* strain as well as on the type of hairy root culture used [16]. In the current study, we used liquid Woody Plant Media (WPM). After 30 days of culture, fresh and dry weights of hairy roots of each of the 5 transformed lines were approximately 81 g·l^−1^ and 8 g·l^−1^, respectively (Figure 1). For each hairy root line, 3 flasks from 3 successive subcultures were utilized. The hairy roots showed stability with regard to a root biomass increase. WPM was the best growth medium for the transgenic roots of other plant species such as *Rhaponticum carthamoides* [21].

### 3.2. Integration and Expression Analysis of the *rolB* Gene

**Figure 2:**
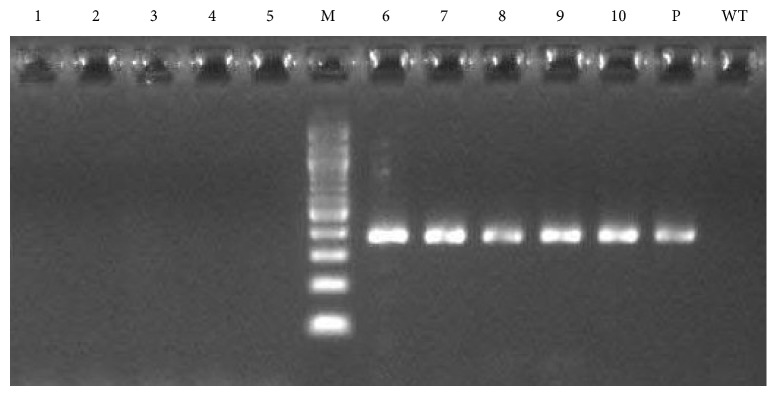
PCR-amplified products of the *rolB* gene in *Lactuca serriola*. Lanes 1–5 show the absence of the *virG* gene in the 5 transgenic hairy root lines. Lanes 6–10 show *rolB* gene fragment amplified in the 5 transgenic hairy root lines. Lane P stands for the plasmid DNA. WT refers to the nontransformed root. Lane M refers to the 100 bp DNA ladder.

**Figure 3:**
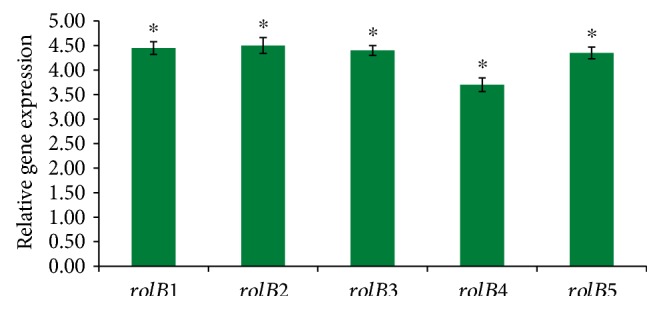
Expression level of the *rolB* gene in the 5 transgenic hairy root lines using real-time qPCR. Data represent three replicates. ^∗^*p* < 0.05 statistically significant.

PCR confirmed *rolB* integration in *Lactuca serriola*, and a fragment of the same size (386 bp) was amplified from each of the 5 survived hairy root lines (Figure 2). A fragment having similar size was also amplified from the plasmid DNA of *A. rhizogenes* AR15834 (positive control). However, no such fragment was amplified from the nontransformed roots (negative control). The *vir*G gene was not amplified from any of the 5 transgenic hairy root lines, confirming the true transformation of hairy roots without *A. rhizogenes* contamination (Figure 2, lanes 1–5). RT-qPCR confirmed *rolB* gene expression in all the 5 survived transgenic lines (Figure 3). The transgenic hairy root lines *rolB1*, *rolB2*, *rolB3*, and *rolB5* exhibited higher levels of *rolB* transcript as compared to *rolB4* line (*p* < 0.05).

### 3.3. Expression Analysis of Flavonoid Biosynthetic Genes

**Figure 4:**
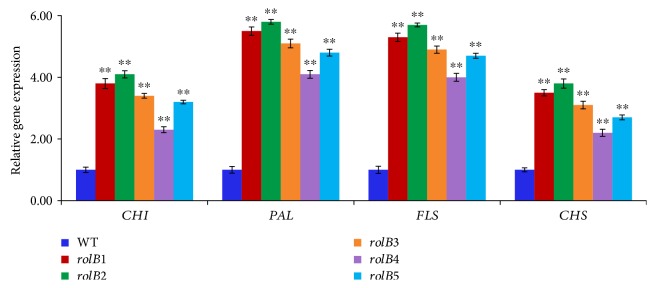
Flavonoid biosynthetic gene expression level in the transgenic roots and nontransformed roots (WT). Data represent three replicates; bars indicate ±standard deviation. ^∗∗^*p* < 0.01 statistically significant.

Analysis of gene expression provides a comprehensive insight into how metabolic pathways regulate flavonoid synthesis in the transgenic and nontransformed roots. RT-qPCR was done to evaluate the expression of 4 flavonoid biosynthetic genes (CHI, PAL, FLS, and CHS) in the transgenic and nontransformed roots. Expression levels of flavonoid biosynthetic genes were higher in the transformed roots as compared to the normal roots (*p* < 0.01; Figure 4). These results could be attributed to the functional role of the *rolB* gene in inducing secondary metabolite biosynthesis in hairy roots by activating their biosynthetic genes. These results are in accordance with those revealed by Chung et al. [11] and Dilshad et al. [27] who found that the expression levels of flavonoid biosynthetic genes in the hairy roots of *Brassica rapa* and *Artemisia carvifolia*, respectively, were higher than those in the normal roots.

### 3.4. Evaluation of Total Phenolic and Flavonoid Content and Total Reducing Power

**Figure 5:**
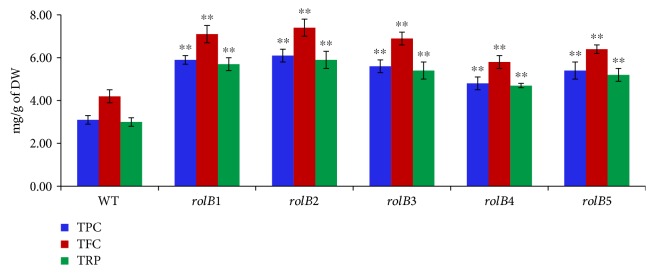
Results of TPC, TFC, and TRP in the 5 transgenic hairy root lines of the *rolB* gene and nontransformed roots (WT). Data are represented in mean ± standard deviation, in milligrams/gram of dry weight (DW). ^∗∗^*p* < 0.01 statistically significant.

TPC results exhibited a 90.3%, 96.7%, 80.6%, 54.8%, and 74.2% increase in the hairy root lines of *rolB1*, *rolB2*, *rolB3*, *rolB4*, and *rolB5*, respectively, compared to the nontransformed root (Figure 5). TFC data revealed a 69%, 76.2%, 64.3%, 38.1%, and 52.4% increase in the hairy root lines of *rolB1*, *rolB2*, *rolB3*, *rolB4*, and *rolB5*, respectively, compared to the nontransformed root (Figure 5). TRP also showed a 90%, 96.7%, 80%, 56.7%, and 73.3% increase in the hairy root lines of *rolB1*, *rolB2*, *rolB3*, *rolB4*, and *rolB5*, respectively, compared to the nontransformed root (Figure 5).

In conclusion, the total flavonoid and phenolic contents and total reducing power were higher in the transformed roots as compared to the normal roots (*p* < 0.01). The results were in accordance with those recorded by Vojin et al. [16] and Ismail et al. [17] who found that the total flavonoid and phenolic contents and total reducing power were higher in the transformed roots as compared to the nontransformed ones of *Lactuca sativa*. Results also showed high significant correlations with the expression levels of *rolB* and flavonoid biosynthetic genes (Table 2). This is the first study that compares the phenolic and flavonoid contents and the corresponding gene expression (CHI, PAL, FLS, and CHS) of transgenic and nontransgenic roots in *Lactuca serriola*.

### 3.5. DPPH Free Radical Scavenging Assay

**Figure 6:**
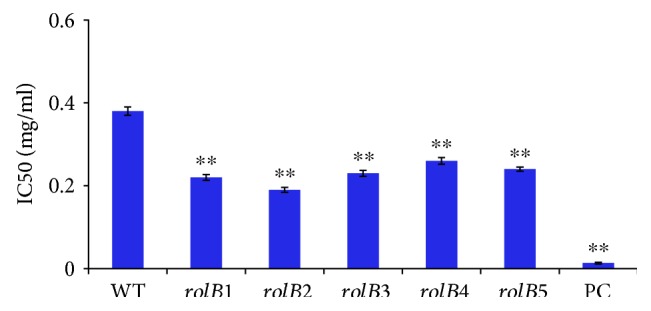
Results of the DPPH assay of the 5 transgenic hairy root lines of the *rolB* gene and nontransformed roots (WT). Data are represented in mean ± standard deviation. PC is a positive control (ascorbic acid). ^∗∗^*p* < 0.01 statistically significant.

The DPPH assay was effective for estimating the enhanced antioxidant potential in the transgenic lines of the *rolB* gene. The extract of *rolB2*-transformed line exhibited the highest radical scavenging capacity (IC50 = 0.19 mg/ml) with a 50% increase as compared to that of the normal roots (0.38 mg/ml) (*p* < 0.01; Figure 6). The extracts of *rolB1*, *rolB3*, *rolB4*, and *rolB5* exhibited a 42.1%, 39.5%, 31.6%, and 36.8% increase as compared to those of the nontransformed roots (*p* < 0.01; Figure 6). This increasing DPPH scavenging potential could be attributed to the high secondary metabolites formed in the transgenic hairy root [17]. The results were in accordance with those recorded by Vojin et al. [16] and Ismail et al. [17].

### 3.6. Cytotoxicity Activity

**Figure 7:**
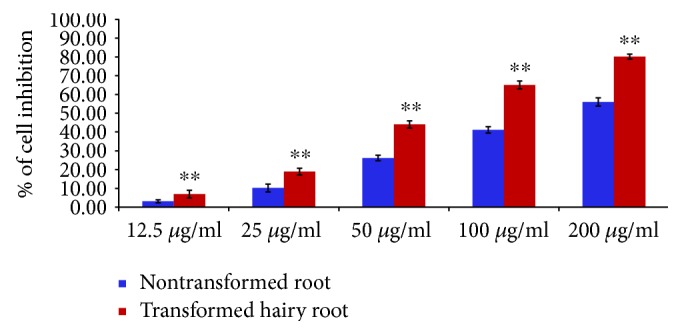
Percentage of cell inhibition of transgenic hairy root and nontransformed root extracts of *Lactuca serriola* against HepG2 human liver cancer cells. Data represent three replicates. ^∗∗^*p* < 0.01 statistically significant.

Screening cytotoxic activity of the extracts of the transgenic and nontransgenic roots against HepG2 human liver cancer cells was investigated. The human cells were subjected to several concentrations of the extracts of the transgenic hairy root *rolB2* line and nontransformed roots. The results showed that the percentage of cancer inhibition relies on the concentration of the extract used (Figure 7). The greater inhibition was recorded at the highest extract concentration (200 *μ*g/ml) (*p* < 0.01; Figure 7), at which the hairy root extracts exhibited 80.21% cancer inhibition whereas the nontransformed root extracts showed 56.02% inhibition. This high cytotoxic activity in hairy roots may be due to the high flavonoid and phenolic content. Our results agreed with various earlier studies which demonstrated that the transgenic roots showed higher antibacterial and cytotoxic activities compared to the nontransgenic roots [28, 29]. In conclusion, the present study suggests that transgenic hairy roots of *Lactuca serriola* could be efficiently used for the antioxidant and medicinal treatments.

## 4. Conclusions

This is the first study that addresses the enhancement of secondary metabolite biosynthesis by hairy root induction in *L. serriola*. The genetic transformation and expression levels of *rolB* in transgenic roots of *Lactuca serriola* were validated by PCR and real-time qPCR analyses. The flavonoid biosynthetic genes (CHI, PAL, FLS, and CHS) exhibited higher levels in the hairy roots than in the nontransformed roots. Hairy roots exhibited significant increases in the total flavonoid and phenolic contents and the total reducing power as compared to the nontransformed roots. Additionally, the cytotoxicity assay revealed that the hairy root extracts exhibited a maximum percentage of 80.21% cancer inhibition whereas the nontransformed root extracts showed a maximum percentage of 56.02% inhibition. The study highlights that the transformation of *Lactuca serriola* with the *rolB* gene may be efficiently used to develop plants with enhanced secondary metabolites and improved medicinally important properties.

## Figures and Tables

**Table 1:** Primers of *rolB* and flavonoid biosynthetic pathway genes used in RT-qPCR analysis.

Gene	Primer sequence (5′–3′)	Reference
*rolB*	F: GCTCTTGCAGTGCTAGATTT	Skała et al. [21]
	R: GAAGGTGCAAGCTACCTCTC	

*vir*G	F: AGTTCAATCGTGTACTTTCCT	Skała et al. [21]
	R: CTGATATTCAGTGTCCAGTCT	

*CHI*	F: TGGTGGCCTAGACAACGATGAGTT	Chung et al. [11]
	R: TCACACTCCCAACTTGGTTTCCCT	

*PAL*	F: AGAACGGTGTCGCTCTTCAG	Chung et al. [11]
	R: TGTGGCGGAGTGTGGTAATG	

*FLS*	F: TTAAAGGAAGGTCTCGGTGGCGAA	Chung et al. [11]
	R: TCATTGGTGACGATGAGTGCGAGT	

*CHS*	F: AGGCTAACAGAGGAGGGTA	Dilshad et al. [27]
	R: CCAATTTACCGGCTTTCT	

*UBQ1*	F: TTCCTTGATGATGCTTGCTC	Chen et al. [22]
	R: TTGACAGCTCTTGGGTGAAG	

**Table 2:** Pearson's correlation among total phenolic and flavonoid contents, total reducing power, DPPH, *rolB*, and flavonoid biosynthetic genes.

	*rolB*	*TPC*	*TFC*	*TRP*	*DPPH*	*CHI*	*PAL*	*FLS*
*TPC*	0.92^∗^	1						
*TFC*	0.90^∗^	0.99^∗∗^	1					
*TRP*	0.90^∗^	0.99^∗∗^	0.99^∗∗^	1				
*DPPH*	−0.80^ns^	−0.95^∗^	−0.95^∗^	−0.96^∗^	1			
*CHI*	0.93^∗^	0.99^∗∗^	0.98^∗∗^	0.99^∗∗^	−0.95^∗^	1		
*PAL*	0.90^∗^	0.99^∗∗^	0.99^∗∗^	0.99^∗∗^	−0.96^∗^	0.99^∗∗^	1	
*FLS*	0.89^∗^	0.99^∗∗^	0.98^∗∗^	0.99^∗∗^	−0.98^∗^	0.99^∗∗^	0.99^∗∗^	1
*CHS*	0.86^ns^	0.99^∗∗^	0.99^∗∗^	0.99^∗∗^	−0.96^∗^	0.98^∗∗^	0.99^∗∗^	0.99^∗∗^

^∗^Significant at *p* < 0.05; ^∗∗^Significant at *p* < 0.01; NS: non significant.
